# Isolated Navicular Algodystrophy and Abnormal Capillaroscopic Pattern in a 50-year-old Smoker: A Possible Microvascular Connection

**DOI:** 10.31138/mjr.280225.era

**Published:** 2025-06-30

**Authors:** Angelo Nigro

**Affiliations:** Department of Rheumatology of Lucania - UOSD of Rheumatology, “Madonna delle Grazie” Hospital, Matera, Italy

**Keywords:** algodystrophy, microcirculation, capillaroscopy, neridronate

## Abstract

We report the case of a 50-year-old female smoker presenting with isolated algodystrophy of the navicular bone and concurrent nailfold videocapillaroscopy (NVC) abnormalities. This observation raises the possibility of an association between microvascular alterations and the etiopathogenesis of algodystrophy. NVC revealed enlarged loops, tortuosity, pericapillary oedema, and microhaemorrhages, findings indicative of impaired microcirculation. Magnetic resonance imaging (MRI) of the foot demonstrated bone marrow oedema consistent with algodystrophy. The patient underwent intramuscular treatment with neridronate, which resulted in full symptomatic remission and radiological resolution at three months. This case highlights the potential role of microvascular dysfunction in algodystrophy and suggests that NVC may serve as a valuable diagnostic tool in patients with unexplained regional pain syndromes. Future studies are required to establish a definitive causal relationship.

## INTRODUCTION

Complex regional pain syndrome type I (CRPS-I), historically referred to as algodystrophy, is a debilitating pain condition characterised by sensory, vasomotor, sudomotor, and motor/trophic disturbances, typically arising in the absence of a defined nerve lesion.^1^ Although the pathogenesis remains incompletely understood, the contribution of vascular and microcirculatory abnormalities has been postulated as a pivotal component.^2^ This report details a case in which micro-vascular dysfunction, as visualised through NVC, was associated with isolated navicular bone algodystrophy. This case is noteworthy for documenting NVC alterations in a patient with localized CRPS-I of the foot, a rarely described phenomenon. It also underscores the relevance of microvascular assessment in the absence of systemic autoimmune pathology. NVC is widely recognised as a critical diagnostic tool in the evaluation of connective tissue diseases, particularly systemic sclerosis, where it enables the detection of characteristic microvascular abnormalities that aid in both diagnosis and disease monitoring.

## CASE REPORT

A 50-year-old Caucasian woman, residing in an urban setting and employed as a housewife, presented with a three-month history of persistent pain, localised oedema, and dysaesthetic skin changes in the right foot. She denied preceding trauma and was not receiving pharmacologic therapy for chronic conditions. The patient had a 20-year history of smoking approximately 20 cigarettes daily. Clinical symptoms included localised skin discoloration, swelling, and joint stiffness, predominantly involving the midfoot.

Extensive immunologic evaluation, including ANA, ENA panel, RF, and anti-CCP antibodies, yielded negative results.^4^ Systemic investigations, including abdominal ultrasound, chest computed tomography, and pulmonary function testing, were unremarkable.

She met the Budapest clinical diagnostic criteria for CRPS-I, fulfilling multiple domains including sensory, vasomotor, sudomotor, and motor/trophic features. Laboratory tests were within physiological ranges, with normal ESR and CRP. MRI of the affected foot demonstrated hyperintense signal alterations on T2 and STIR sequences confined to the navicular bone, consistent with marrow oedema and early algodystrophic changes.^5 , 6^

Given the patient’s recurrent episodes of acrocyanosis, periungual NVC was performed as a targeted vascular assessment. Imaging was acquired using the Capillary.io platform at 250× magnification and interpreted both via its integrated algorithm CAPI-detect and by an expert physician trained in capillaroscopy.^7^ NVC findings included disorganized architecture with enlarged capillary loops, tortuosity, oedema in the pericapillary space, and microhaemorrhage events.

## TREATMENT AND FOLLOW-UP

The patient was initiated on intramuscular neridronate therapy at a cumulative dose of 400 mg, administered as 25 mg over 16 alternate days.^8^ Over subsequent weeks, a progressive attenuation of symptoms was observed. At the three-month follow-up, she exhibited complete clinical remission, and repeat MRI revealed resolution of the previously noted bone marrow oedema.^9^

**Figure 1.**
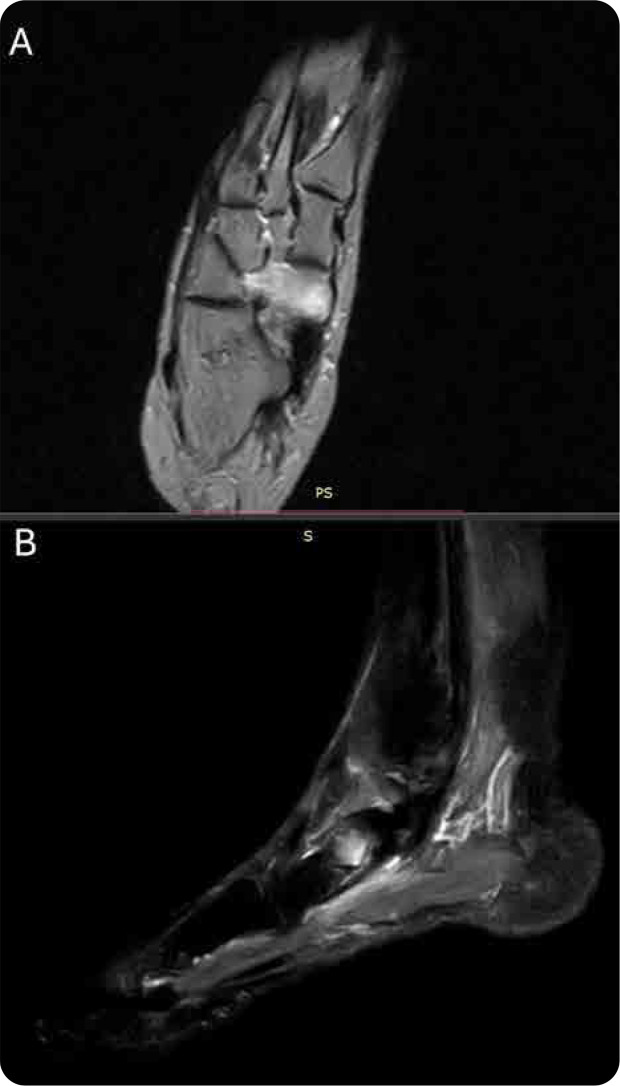
MRI images of the foot with T2-weighted (A) and STIR (B) sequences showing bone marrow oedema involving the entire navicular bone of the right foot.

**Figure 2.**
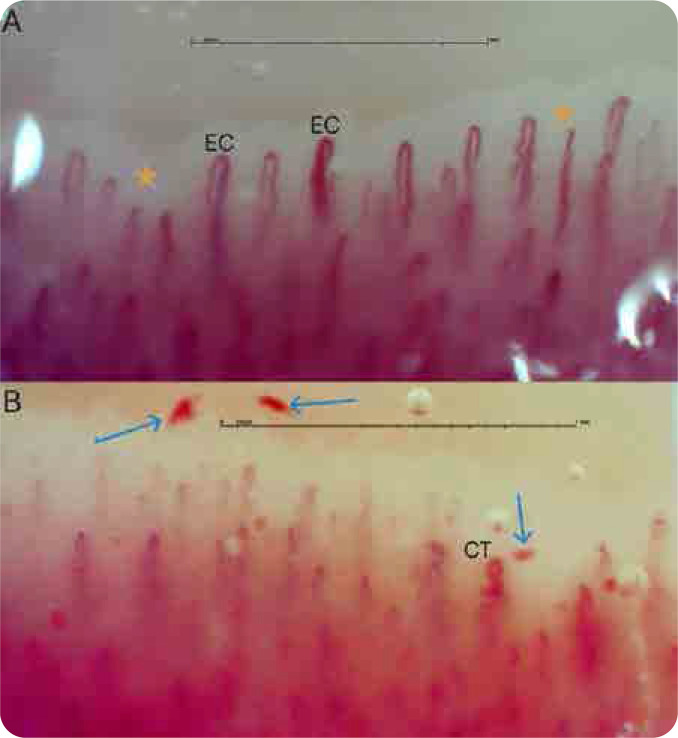
Capillaroscopic images obtained from the fourth finger of the left hand (**A**) and the fourth finger of the right hand (**B**). Panel A shows enlarged capillaries (EC) and pericapillary oedema (asterisk), while Panel B reveals microhaemorrhages (arrows) and capillary tortuosity (CT).

Follow-up nailfold videocapillaroscopy (NVC) revealed persistent microvascular abnormalities, despite complete resolution of clinical symptoms and radiological findings. This enduring capillary morphological alteration prompts a refined pathophysiological interpretation, suggesting an underlying chronic vasculopathic endotype, potentially driven by sustained tobacco-related endothelial injury. Extended longitudinal monitoring beyond 6 to 12 months appears crucial to elucidate the trajectory, clinical significance, and prognostic implications of these capillaroscopic findings.

## DISCUSSION

The literature supports an association between micro-vascular dysregulation and CRPS-I; however, objective in vivo evidence via NVC analysis remains limited.^1,5^ In the present case, abnormal NVC morphology lends credence to the hypothesis that endothelial dysfunction may contribute to the initiation or perpetuation of algodystrophic processes.^3, 6^ Structural microangiopathic changes, such as capillary dilatation, tortuosity, and pericapillary leakage, can compromise tissue perfusion, promote local hypoxia, and trigger nociceptive sensitisation through neurogenic inflammation.

The decision to initiate neridronate was guided by data demonstrating its efficacy in CRPS-I, likely attributable to its dual antiresorptive and anti-inflammatory mechanisms.^2,8^ Notably, smoking cessation was not achieved during the treatment course, a factor that may have perpetuated vascular abnormalities and endothelial stress.^7^

The NVC findings observed in this patient differ markedly from those typically seen in systemic autoimmune diseases such as systemic sclerosis, where megacapillaries and avascular areas are commonly present. In contrast, the pattern in this case is more consistent with a smoking-related microvascular insult, characterised by diffuse pericapillary oedema and microhaemorrhages, while overall capillary density remains preserved.

In light of these observations, the integration of NVC into both the initial diagnostic assessment and longitudinal monitoring of CRPS-I may offer substantial clinical utility, particularly in patients with known vascular risk factors. Further studies are necessary to define its prognostic significance and therapeutic implications. Limitations of this report include its single-case nature and the lack of extended longitudinal NVC monitoring. Hence, definitive conclusions regarding the temporal relationship between microvascular dysfunction and CRPS-I cannot be drawn.

## CONCLUSION

This case reinforces the potential pathogenic role of microvascular dysfunction in CRPS-I and highlights the utility of NVC in selected clinical contexts. In individuals with atypical algodystrophy and concurrent vascular risk profiles, such as chronic tobacco use, NVC evaluation may uncover clinically silent endothelial injury. The persistent abnormalities observed in this patient, despite symptom resolution, point toward a chronic vascular phenotype and a complex interplay between vascular and nociceptive mechanisms.^9^ Elucidating this relationship may provide future avenues for risk stratification and targeted therapeutic strategies. Furthermore, NVC may represent a valuable, non-invasive modality for longitudinal surveillance in comparable clinical scenarios, particularly among individuals with heightened vascular risk profiles, including chronic smokers.

## CONSENT FOR PUBLICATION

Written informed consent was obtained from the patient for publication of this case report.

## COMPETING INTERESTS

The author declares that they have no competing interests.

## FUNDING

No funding was received for this study.

## AUTHORS’ CONTRIBUTIONS

The author performed all contributions to the case report.
